# Efficacy and safety of a music-therapy facilitated pulmonary telerehabilitation program in COPD patients: the COPDMELODY study protocol

**DOI:** 10.3389/fmed.2024.1361053

**Published:** 2024-03-08

**Authors:** Minghui Shi, Lulu Yang, Shiwei Qumu, Jieping Lei, Ke Huang, Ruoxi He, Hongtao Niu, Fen Dong, Siyuan Wang, Jiaze He, Ting Yang

**Affiliations:** ^1^National Center for Respiratory Diseases, Beijing, China; ^2^Institute of Respiratory Medicine, Chinese Academy of Medical Sciences, Beijing, China; ^3^National Clinical Research Center for Respiratory Diseases, Beijing, China; ^4^Department of Pulmonary and Critical Care Medicine, Center of Respiratory Medicine, China-Japan Friendship Hospital, Beijing, China; ^5^Capital Medical University, Beijing, China; ^6^Fangzhuang Community Health Service Center, Capital Medical University, Beijing, China; ^7^Department of Clinical Research and Data Management, Center of Respiratory Medicine, China-Japan Friendship Hospital, Beijing, China; ^8^Department of Rehabilitation Medicine, China-Japan Friendship Hospital, Beijing, China

**Keywords:** chronic obstructive pulmonary disease, telerehabilitation, music therapy, randomized controlled trial, pulmonary rehabilitation

## Abstract

Despite considerable evidence for the benefit in chronic obstructive pulmonary disease (COPD), the implementation of pulmonary rehabilitation (PR) is insufficient. However, music therapy may help address this gap due to its unique benefits. Therefore, we aimed to develop a music-therapy facilitated pulmonary telerehabilitation program based on rhythm-guided walking, singing, and objective telemonitoring. A supervised, parallel-group, single-blinded, randomized controlled clinical trial will be conducted, including 75 patients with COPD anticipated to be randomized in a 1:1:1 ratio into three groups. The intervention groups will receive a 12-week remotely monitored rehabilitation program, while the usual care group will not receive any rehabilitation interventions. Of the two intervention groups, the multi-module music therapy group will contain rhythm-guided walking and singing training, while the rhythm-guided walking group will only include music tempo-guided walking. The primary outcome is the distance of the incremental shuttle walking test. Secondary outcomes include respiratory muscle function, spirometry, lower extremity function, symptoms, quality of life, anxiety and depression levels, physical activity level, training adherence, and safety measurements. The results of this study can contribute to develop and evaluate a home-based music-facilitated rehabilitation program, which has the potential to act as a supplement and/or substitute (according to the needs) for traditional center-based PR in patients with stable COPD.

**Clinical trial registration**: https://classic.clinicaltrials.gov/, NCT05832814.

## 1 Introduction

Chronic obstructive pulmonary disease (COPD) is the leading cause of morbidity and premature mortality globally, surpassing cancer, with the third-highest number of deaths in China, resulting in overwhelming pressure on healthcare systems (1). Moreover, living with COPD is often a daily struggle marked by reduced physical activity, further increasing disease progression, and creating a vicious circle (2). Psychologically, patients with COPD tend to suffer from increased stress and anxiety due to dyspnea and muscle dysfunction (3, 4).

Despite significant evidence on exercise capacity, symptoms and prognosis, the implementation of conventional pulmonary rehabilitation (PR) for COPD is insufficient, mainly due to inadequate resources, high cost, travel distance, lack of time, and low self-efficacy (5). Nonetheless, various telerehabilitation programs have been developed to overcome these barriers (6, 7). However, they play a limited role in reversing patients’ low interests in exercises and rejection of rehabilitation due to fear of dyspnea and fatigue during training. Moreover, it is difficult to ensure the exact intensity of home-based exercises without professional equipment and real-time supervision (8, 9) and telerehabilitation programs with minimal equipment support are limited.

Recent studies have highlighted that music-facilitated rehabilitation not only benefits the interrelated physical and psychological consequences of patients but also improves motivation and training adherence. Music therapy (MT) targeting COPD includes passive (listening to music during aerobic exercises) and active elements (singing) (10, 11). Using rhythms to control the walking speed could offer an easy, economical way to ensure the exercise intensity at home. It can also be regarded as distractive auditory stimulus therapy, helping with dyspnea and fatigue during exertion (12). Additionally, singing promotes adaptation to breath control and training related respiratory muscles with less cost and more interest (13). Therefore, integrating tele-PR and MT may help overcome the previously mentioned barriers. However, there is a lack of relevant home-based studies as most of the existing studies were conducted in the hospital or community. Moreover there is limited evidence on the integrated effects of singing and music-guided aerobic exercises, with most studies only applying one kind of intervention (14).

Therefore, to identify a helpful and easy-to-use rehabilitation mode, our study aimed to develop a home-based, music-facilitated rehabilitation program for patients with COPD, using music tempo-guided walking exercises and breathing exercises, such as singing. Based on a thorough literature review, this is the first study to combine the two forms of MT for home-based PR and apply objective recording and supervision of training implementation using wearable sensors (a sports wristwatch that monitors step counts and walking distance) for music-facilitated tele-PR. This study protocol describes the process to investigate the efficacy and safety of the developed MT-facilitated telerehabilitation program (including rhythm-guided walking and singing).

The primary aim of this study is to explore the efficacy of this home-based, MT- facilitated rehabilitation program (including rhythm-guided walking and singing). The secondary aim is to explore whether addition of singing training results in improvements compared with rhythm-guided walking alone. Therefore, we hypothesize that (1) our MT-facilitated tele-PR mode could benefit patients with COPD in terms of exercise capacity, respiratory musculature, and symptoms; and (2) singing training could result in greater improvement compared with rhythm-guided walking alone.

## 2 Methods and analysis

### 2.1 Study design

**Figure 1:**
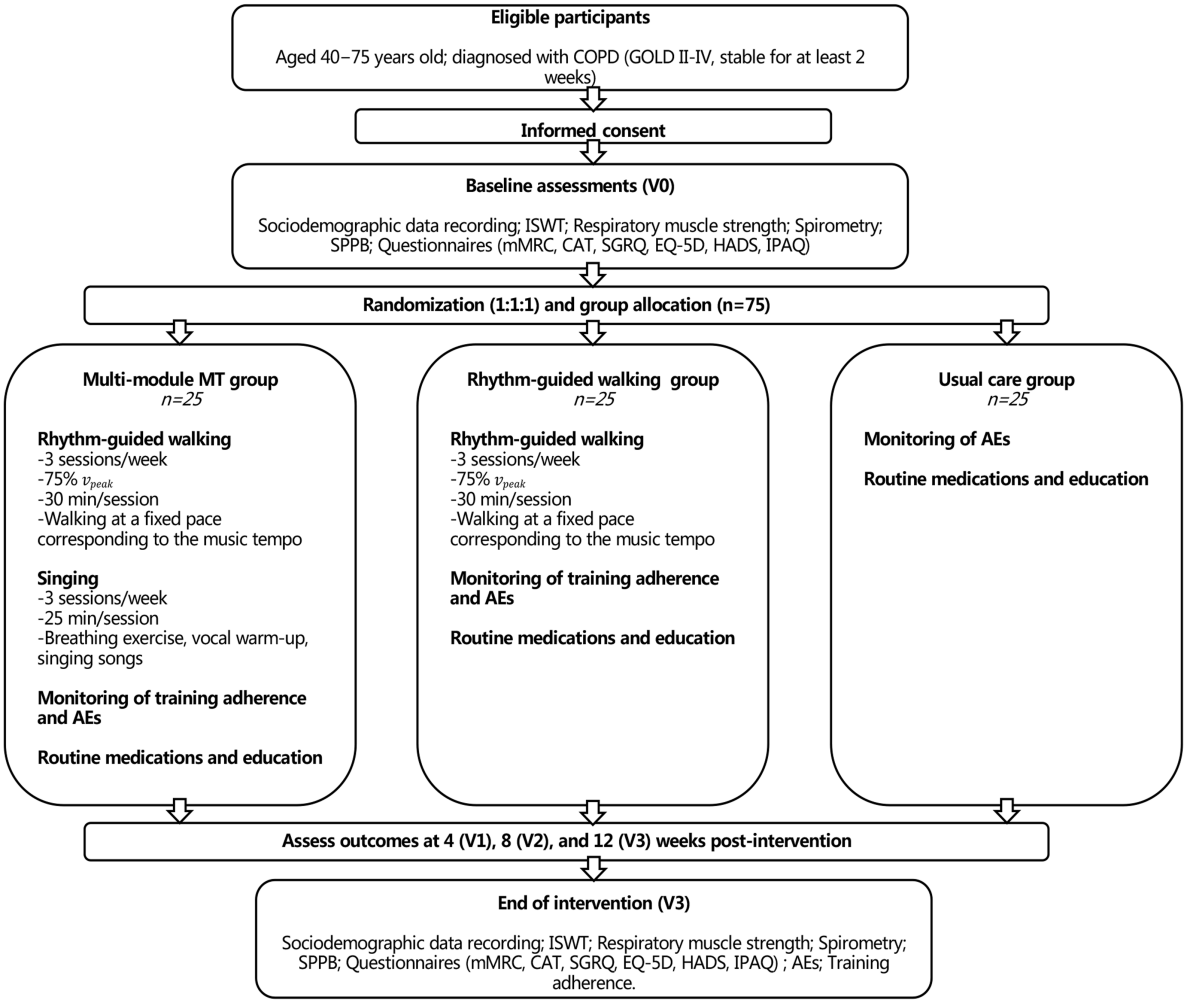
Flow diagram of study design.

This study will use a multi-center, prospective, three-arm, randomized controlled trial design. The study will include patients with COPD enrolled in a multi-module MT rehabilitation group (multi-module MT group), a rhythm-guided walking rehabilitation group (rhythm-guided walking group), and a usual care group. The study period will last for 12 weeks, and the outcomes will be measured at baseline (V0), 4 weeks (V1), 8 weeks (V2), and the end of intervention (V3). A Consolidated Standards of Reporting Trials (CONSORT) flow diagram is illustrated in Figure 1. The study protocol will be reported according to the Standard Protocol Items: Recommendations for Intervention Trials 2013 (SPIRIT) (15) guidelines and the intervention procedures are described according to the CONSORT 2010 Checklists (16). The trial is registered at ClinicalTrials.gov (registration number: NCT05832814). The study schedule and assessments are summarized in Table 1.

**Table 1:** Summary of study schedule.

	Enrolment	Baseline (V0)	4 weeks (V1)	8 weeks (V2)	12 weeks (V3)
Eligibility screening					
Informed consent	√				
Randomization		√			
Allocation		√			
Interventions					
Multi-module music therapy		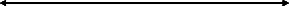			
Rhythm-guided walking		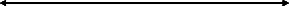			
Usual care		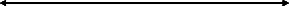			
Assessments					
Demographic characteristics		√			
ISWT		√	√	√	√
MIP; MEP		√	√	√	√
Spirometry		√			√
SPPB		√	√	√	√
mMRC; CAT		√	√	√	√
SGRQ; EQ-5D		√	√	√	√
HADS		√	√	√	√
IPAQ		√	√	√	√
Training adherence			√	√	√
AEs			√	√	√

ISWT, incremental shuttle walking test; MIP, maximal inspiratory pressure; MEP, maximal expiratory pressure; SPPB, Short Physical Performance Battery; mMRC, Modified Medical Research Council; CAT, COPD assessment test; SGRQ, St. George’s Respiratory Questionnaire; EQ-5D, EuroQoL-5D Questionnaire; HADS, Hospital Anxiety and Depression Scale; IPAQ, International Physical Activity Questionnaire; AE, adverse event.

### 2.2 Study population and recruitment

This multi-center study will be conducted at the China-Japan Friendship Hospital. Patients will be recruited from the China-Japan Friendship Hospital, Beijing Tiantan Hospital, Beijing Luhe Hospital, and the Second Affiliated Hospital of Xi’an Jiaotong University, Qingdao Municipal Hospital. Advertising strategies will include flyers within the hospital and professional recommendations. Potential eligible patients who are interested in participation will be invited to have either a face-to-face or telephone meeting wherein the researchers will explain the study in detail and allow time for questions. Potential patients will be asked about medical history and undergo a pulmonary function test to ensure final eligibility. The inclusion and exclusion criteria are summarized in Table 2. Patients who meet the eligibility criteria will be invited to sign informed consent forms and complete baseline outcome assessment.

**Table 2:** Inclusion and exclusion criteria.

Inclusion criteria	Exclusion criteria
1. Adults aged 40–75 years	1. Acute myocardial infarction within 4 weeks, unstable angina, uncontrolled atrial or ventricular arrhythmia, and heart failure
2. Diagnosed with COPD of GOLD II-IV	2. Hypertrophic cardiomyopathy, severe valvular heart disease, severe aortic stenosis
3. Current outpatient, stable for at least 2 weeks prior to the intervention enrolment	3. Acute pulmonary embolism, uncontrolled asthma, respiratory failure
4. Able to use a smartphone	4. Comorbidity precluding exercise training (e.g., orthopedic, neurological, or cognitive conditions)
5. Able to understand the purpose of the clinical trial and voluntarily participate with signed informed consent	5. Presence of malignant tumor
	6. Involved in pulmonary rehabilitation programs within the past 12 months

### 2.3 Randomization, allocation concealment, and blinding

All patients will be randomized in a 1:1:1 ratio (adhering to block randomization within each center) into three groups: Multi-module MT, rhythm-guided walking, and usual care groups using a table of random numbers generated in the SPSS statistical package held by an independent statistical analyst. Group information will be stored in an opaque envelope and sealed thereafter. After obtaining informed consent and baseline assessment, the study coordinator will unseal the envelope to obtain the random numbers and grouping information for each participant.

Due to the nature of the intervention, neither the participants nor the exercise instructors will be blinded. However, pulmonology and rehabilitation nurses conducting outcome assessments and statistical analysts will be blinded to the assignments.

### 2.4 Interventions

All patients will continue taking routine medications, such as bronchodilators and steroid inhalers, according to their respective conditions and will maintain their regular treatment visits throughout the study period. Additionally, patients in the two intervention groups will receive two different forms of PR programs conducted by a multi-disciplinary team consisting of pulmonologists, rehabilitation physicians, pulmonology and rehabilitation nurses, music therapists, and research assistants (Tables 3, 4). The 12-week PR program is based on exercise, facilitated with music.

**Table 3:** Multi-module MT rehabilitation program.

Aerobic training	
Rhythm-guided walking	Exercise at an intensity of 75% of the patients’ peak speed evaluated by ISWT
	Walking at a fixed pace following the music tempo matched with the targeted speed
Duration	30 min
**Respiratory muscle training**	
Breathing exercise Vocal warm-up Singing songs	Breathing control, pursed lips breathing, and abdominal breathing Introducing ‘primal sounds’ such as Hey, Ho, Ha, etc. Choosing appropriate songs fit for patients in terms of phrase lengths, breath points, lyrics, melodic challenge, and range
Duration	25 min

MT, music therapy; ISWT, incremental shuttle walking test.

**Table 4:** Rhythm-guided walking rehabilitation program.

Aerobic training	
Rhythm-guided walking	Exercise at an intensity of 75% of the patients’ peak speed evaluated by ISWT
	Walking at a fixed pace following the music tempo matched with the targeted speed
Duration	30 min

ISWT, incremental shuttle walking test.

Home-based rehabilitation prescriptions will be conveyed by a software installed on the smartphone, containing different function modules based on the intervention group. Each patient will be provided a secure user account to log into the software.

#### 2.4.1 Multi-model MT group

Participants in the multi-model MT group will undergo two types of training: rhythm-guided walking (aerobic exercises) and singing (respiratory muscle training). Additionally, their softwares will contain two function modules: the “rhythm-guided walking” module which includes melodies with various rhythms (60–120 bpm) that can match individual walking speeds and the “singing” module which includes various songs suitable for patients to sing, which are selected and recorded by music therapists.

The participants will undertake three walking sessions of at least 30 min per week. The exercise intensity (in the form of walking speed) will be prescribed individually, according to the ISWT results. The peak walking speed ( $v_{\mathrm{peak}}$ ) will be assessed by the ISWT, and the targeted training speed will be set at 75%, according to the American College of Sports Medicine. A music rhythm (i.e., stride frequency) matching the target speed will be calculated using the following formula:


(1) $$\begin{array}{l} \mathrm{Stride}\;\mathrm{frequency}\;\left(\mathrm{steps}\cdot \min^{-1}\right)=0.75\times v_{\mathrm{peak}}\;\left(\mathrm{km}\cdot h^{-1}\right)\\ \times 100\times \mathrm{steps}\;\mathrm{per}\;\mathrm{shuttle}/60 \end{array}$$

(2) $$\begin{array}{l} \mathrm{Music}\;\mathrm{rhythm}\;\left(\mathrm{beats}\cdot \min^{-1}\right)\\ =\mathrm{stride}\;\mathrm{frequency}\;\left(\mathrm{steps}\cdot \min^{-1}\right) \end{array}$$

Patients will be asked to follow this individualized music rhythm and walk at a fixed pace to maintain a constant speed. During the aerobic exercise, patients will need to open the “rhythm-guided walking” module on the software, to play the melodies with the prescribed specific tempo on a loop. Patients will need to regulate their stride frequency corresponding to the tempo to achieve adequate exercise intensity. During this, they will be asked to wear a sports wristwatch displaying their walking distance.

Additionally, the participants will undertake three singing sessions of at least 25 min per week, following the prescriptions conveyed by the software. One session includes 10 min of breathing exercises and vocal warm-up with a focus on awareness of supporting musculature during inhalation and exhalation, as well as subconscious vocal release before singing songs. It rounds up with 15 min of singing songs to improve the strength, endurance, and flexibility of the respiratory muscles. During the respiratory muscle training, patients need to open the “sing” module to follow the guiding audios and videos.

#### 2.4.2 Rhythm-guided walking group

Participants in the rhythm-guided walking group will perform only music-facilitated walking exercises as previously described in the multi-module MT group.

#### 2.4.3 Usual care group (waiting-list control group)

During the study period, patients in the usual care group will not receive any rehabilitation intervention during the 12-week study period. However, they will be invited to access the music-facilitated PR program once the follow-up test is complete, thus, establishing a waiting-list control group.

### 2.5 Outcome measures

Table 1 illustrates the items to be measured and the time window for data collection. The primary outcome is the exercise capacity measured by ISWT (relative change in the ISWT distance seen at 4-, 8-, and 12-weeks post-intervention compared with baseline) in all three subgroups. The ISWT will be determined according to the recommendations of the European Respiratory Society/American Thoracic Society (17). In addition, as an externally paced maximal exercise test, walking speed will be controlled by a series of pre-recorded signals. The walking speed increases progressively until the participant can no longer continue.

The secondary outcomes are:

1. Respiratory muscle function: This will be assessed by the maximal inspiratory and expiratory pressures using the Gio Digital Pressure Gauge (18).
2. Pulmonary function test: This will be assessed by spirometry performed using automated equipment as the guideline (19).
3. Lower Extremity Function: This will be assessed using the Short Physical Performance Battery (SPPB), an objective tool for measuring lower extremity physical performance status. The SPPB is based on three timed tasks: standing balance, walking speed, and chair-stand tests (20).
4. Symptoms: Chronic activity-related dyspnea will be assessed using the modified Medical Research Council dyspnea scale (mMRC). The respiratory health status will be assessed using the COPD Assessment Test (CAT).
5. Health-related quality of life (HRQoL): This will be measured using St. George’s Respiratory Questionnaire (SGRQ) (21, 22) and the EuroQoL-5D Questionnaire (EQ-5D) (23). These are simple, generic HRQoL instruments widely used as patient-reported outcome measures.
6. Anxiety/depression rates: This will be evaluated in the hospital using the Hospital Anxiety and Depression Scale (HADS) which comprises seven items each for the anxiety and depression subscales. The HADS is a validated measure for assessing anxiety and depression symptoms and is recommended for patients with COPD (24, 25).
7. Physical Activity (PA) level: Daily PA will be measured using the International Physical Activity Questionnaire (IPAQ) adopted in Chinese, which presents acceptable reliability and high repeatability values (26).
8. Training adherence: Patient training adherence is defined as the percentage of the total number of completed training sessions. The supervising hospital staff will record patient adherence.
9. Safety measurements: All adverse events (AEs) that occur during the study will be recorded and evaluated for relevance to the intervention. AEs include exacerbations, exercise injuries, and falls.

### 2.6 Data management and quality control

Patients will be required to practice rhythm-guided walking or singing until they master it on their first hospital visit. Telephone calls or video conferences by rehabilitation instructors will be integrated at the start of the program to ensure proper execution. After each training session, participant performance will be automatically transferred to the digital platform to facilitate the identification and verification of adherence to the prescribed exercise plan. Research assistants will verify training completion every day, record training adherence, and remind the patients to exercise.

To decrease measurement error, each participant will be assessed by a single nurse during different visits. Study data will be maintained on a password-protected platform and backed up to a secure external hard drive, with access restricted to authorized researchers and staff. Automatic plausibility controls will be set to detect any inconsistencies or inaccuracies during data entry.

### 2.7 Statistical analysis

All statistical analyses will be performed using full intention-to-treat analyses, with scores on the dependent variables for dropouts carried-over using the multiple imputation method. Independent samples *t*-tests, chi-square tests, and F-tests will be used to analyze differences between dropouts and completers regarding baseline characteristics.

All three groups will be examined using a two-sample Student’s *t*-test for normal distributions or a Wilcoxon test for non-normal distributions (pre and postscores). The differences among the three groups at each time point (4, 8, and 12 weeks) will be analyzed using the Student’s *t*-test or Mann–Whitney U test. A two-way analysis of variance with repeated measures will be used to determine the effects of time and group on the dependent variables.

All data will be analyzed using SPSS V.21.0 (IBM) software packages. Statistical significance is set at two-sided *p* < 0.05.

#### 2.7.1 Sample size calculation

Considering the lack of previous studies combining music-guided exercise with singing, we did not perform a formal sample size calculation. Nevertheless, we calculated a sample size based on relevant studies applying similar rehabilitation interventions (27, 28). We used ISWT (the primary outcome) to calculate the sample size, as exercise capacity was one of the most sensitive outcome measures to detect the benefit of rehabilitation. We assumed that the mean changes in ISWT of multi-model MT group, rhythm-guided walking group and usual care group are 40 m, 27 m, and -15 m, respectively (27, 28), and the combined standard deviations (SD) are 35 m, 38 m, and 40 m, respectively. Based on a rate of type-I error of α = 0.05, a power of 80% (rate of type-II error of β = 0.1), and a possible dropout rate of 50%, each group will require at least 18 patients.


$$n=\frac{\psi^{2}\left(\frac{\sum S_{i}^{2}}{k}\right)}{\sum \left(\bar{X_{i}}-\bar{X}\right)\;\left(k-1\right)}$$

Therefore, 75 participants are anticipated to be enrolled into this study.

## 3 Discussion

This trial aims to establish a music-facilitated telerehabilitation program using wearable sensors and web applications for COPD. Unlike common telerehabilitation modes, the program creatively includes music tempo-guided PA and breathing exercises in the form of singing, to develop a helpful and attractive rehabilitation mode.

Supplementing tele-PR with music therapy is the main feature of this study. Compared to traditional aerobic exercise, music-guided endurance training can help patients exercise more by decreasing or delaying the perception of exercise-induced dyspnea and fatigue (12). Additionally, music-guided respiratory training (i.e., singing) could help patients learn how to control respiratory function, including training and coordinating the related inspiratory and expiratory musculature (29) in a better psychological status. Moreover, MT is indicated to help patients overcome the fear of dyspnea and fatigue related to physical activity, thereby improving their adherence to PR.

Notably, various parts of PR may play distinct roles in benefiting patients’ functions. For example, systematic endurance training can significantly improve exercise capacity through its direct effect on peripheral muscles (30, 31), while respiratory training may contribute more to correct the imbalance between respiratory muscle function and load and relieve dyspnea (32). Similar to the relationship between conventional physical exercise and respiratory training, music-guided endurance exercise combined with singing training has the potential to provide additional benefits through various physiological mechanisms compared to individual applications. Unlike most relevant studies which focus on only one kind of MT intervention, our research designed a home-based rehabilitation program with the integration of music-guided endurance exercise and singing training to maximize the clinical benefits. Additionally, by comparing the difference between the two intervention groups, we aim to offer more evidence on whether singing could bring additional improvements compared with rhythm-guided walking alone, especially in respiratory muscle function.

As a country with a large territory and population, China has an unequal healthcare system. Moreover, there is high variability in access to PR, which means that PR is almost absent in primary healthcare institutions, leaving a wide gap between numerous patients with COPD and limited rehabilitation resources (33). However, recent guidelines support the development of home-based PR, telehealth, and mHealth interventions in which objective, timely, and reliable monitoring of treatment implementation is deemed important. Remote technologies and wearable activity trackers could strengthen contact with patients, increase participation rates, and long-term adherence to healthy behaviors (34). Furthermore, although the digital literacy of patients with COPD is considered a barrier to PR participation, data from a recent study revealed encouraging results with the successive use of smartphones and wearable technology by an older respiratory population (35).

Therefore, there is an urgent need for innovative, effective, and safe PR strategies with easier accessibility and better compliance. The results of this study can contribute to develop and evaluate a home-based music-facilitated rehabilitation program, which has the potential to function as a supplement and/or substitute (according to the needs) for traditional center-based PR in patients with stable COPD. However, undoubtedly, barriers to clinical research will ensue during the program’s implementation stage. Nonetheless, we have designed the study to minimize such barriers. First, convincing participants to comply on the visit plan across the 12-week period is a potential challenge, especially for the usual care group. Therefore, we will inform the patients that those in the control group will also be given the opportunity to receive the same intervention upon completion of the study. For patients in the intervention groups, researchers will confirm the exercise performances every day through the platform and provide reminders when necessary. Additionally, for our secondary aim, we may not be able to detect statistical significance in distance of ISWT when comparing the multi-model MT group with the rhythm-guided walking group in this pilot study, however, we do expect a better respiratory muscle function and mental health which are more sensitive to the addition of singing training.

## Ethics statement

The studies involving humans were approved by the institutional review board at the China-Japan Friendship Hospital. The studies were conducted in accordance with the local legislation and institutional requirements. The participants provided their written informed consent to participate in this study.

## Author contributions

MS: Writing – review & editing, Writing – original draft. LY: Writing – review & editing, Conceptualization. SQ: Writing – review & editing, Conceptualization. JL: Writing – review & editing, Methodology. KH: Writing – review & editing, Conceptualization. RH: Writing – review & editing. HN: Writing – review & editing. FD: Writing – review & editing. SW: Writing – review & editing. JH: Writing – review & editing. TY: Writing – review & editing, Writing – original draft.
